# Data from crosslinked PS honeycomb thin film by deep UV irradiation

**DOI:** 10.1016/j.dib.2015.11.012

**Published:** 2015-11-17

**Authors:** Van-Tien Bui, Hwa Su Lee, Jae-Hak Choi, Ho-Suk Choi

**Affiliations:** a Department of Chemical Engineering, Chungnam National University, 220 Gung-Dong, Yuseong-Gu, Daejeon 305-764, Republic of Korea; b Polymer Science and Engineering, Chungnam National University, 220 Gung-Dong, Yuseong-Gu, Daejeon 305-764, Republic of Korea

**Keywords:** Ordered structure, Robust honeycomb film, “Disc-in-pore”, Topology, Improved phase separation, Polystyrene

## Abstract

Thin polystyrene (PS) films with highly ordered honeycomb pattern were successfully fabricated by an improved phase separation method. The PS film was successfully crosslinked after applying a deep UV irradiation. This work presents a proof of crosslinking PS by characterizing ATR-FTIR, TGA and the wetting property of the honeycomb films, which were prepared using a solvent/non-solvent ratio of 90/10, before and after 6 h of UV irradiation.

**Specifications Table**

**Table:** 

Subject area	Physics, Chemistry
More specific subject area	Polymer honeycomb patterning film
Type of data	Table, figure, image (scanning electron microscopy)
How data was acquired	Large-scale production of the patterning film was proved by the photograph of sample. The introduction of oxygen-containing functional groups was confirmed through examining the Fourier transform-infrared (FTIR) spectra. The thermal property of the honeycomb film was characterized using TGA. The “disc-in-pore” topology was verified from SEM image. The wetting properties of honeycomb film were characterized by measuring water contact angle using a drop-shaped analyzer (Krüss DSA 100, Germany).
Data format	Analyzed, tabulated and plotted
Experimental factors	Chloroform/methanol volume ratio, humidity, temperature
Experimental features	The PS honeycomb film was prepared with chloroform/methanol ratio of 90/10 under ambient air environment (i.e. RH of 55% and temperature of 25 °C). The UV irradiation was lasted for 6 h.
Data source location	Chungnam National University, Deajeon, South Korea
Data accessibility	Data is provided with this article

**Value of the data**

• The photograph of the sample represents the possibility of large-scale, easy production of this method.
• ATR-FTIR and TGA analysis indicate that PS honeycomb film was successfully crosslinked after applying a deep UV irradiation.
• Water contact angle measurement is very simple and useful for characterizing the functionalized surface.

## 1 Experimental design, materials and methods

Experimental details are described in Ref. [1]. The honeycomb PS films used in this work were prepared with chloroform/methanol ratio of 90/10, and under ambient air environment.

**Fig. 1:**
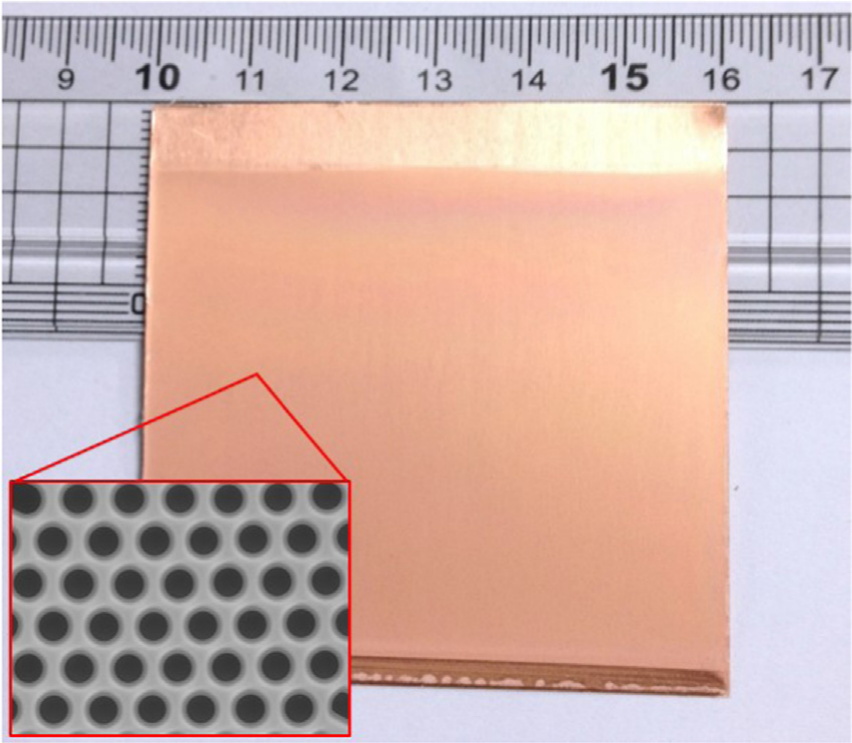
Photograph of a large PS film coated onto a copper substrate. The inset presents the highly ordered honeycomb structure of the PS film.

The photograph presented in Fig. 1 demonstrates the potential for the proposed strategy to be expanded to large-scale production of honeycomb micropatterned films. By using the 2-step method, the large-scale polymer film with uniform thickness was firstly fabricated by taking advantages of a bar-coating technique. In comparision, the drop-casting technique, which is usually used in preparing honeycomb film by a BF method [2], [3], [4], [5], [6], is difficult in fabricating the uniform film, especially large-scale film, because the surface tension of the solution causes the lens shape of the droplet. The homogeneous sample color reflects the high uniformity of the patterned film, which was observed throughout the coated surface (~30 cm^2^). For more insight into the geometry, the inset is SEM image, which demonstrates the highly ordered honeycomb structure of the PS film.

**Fig. 2:**
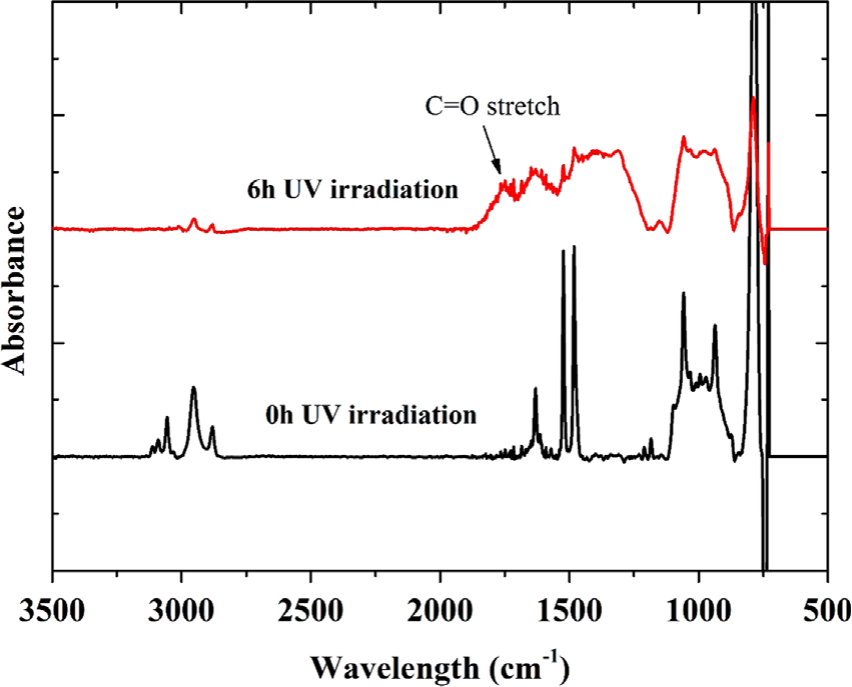
ATR-FTIR spectra of the PS honeycomb films before and after the 6 h UV irradiation.

Fig. 2 shows the ATR-FTIR spectra of the PS honeycomb films before and after the 6 h UV irradiation. The aromatic C–C bonds and C–H bending vibrations of the benzene ring include three peaks of 1600, 1500, and 1400 cm^−1^ [7]. After 6 h of UV irradiation, a broad band at 1750 cm^−1^ appeared and it can be assigned as a C=O stretch of ketone groups. Thus, it indicates that oxygen was introduced into polymer matrix to form oxygen-containing functional groups.

**Fig. 3:**
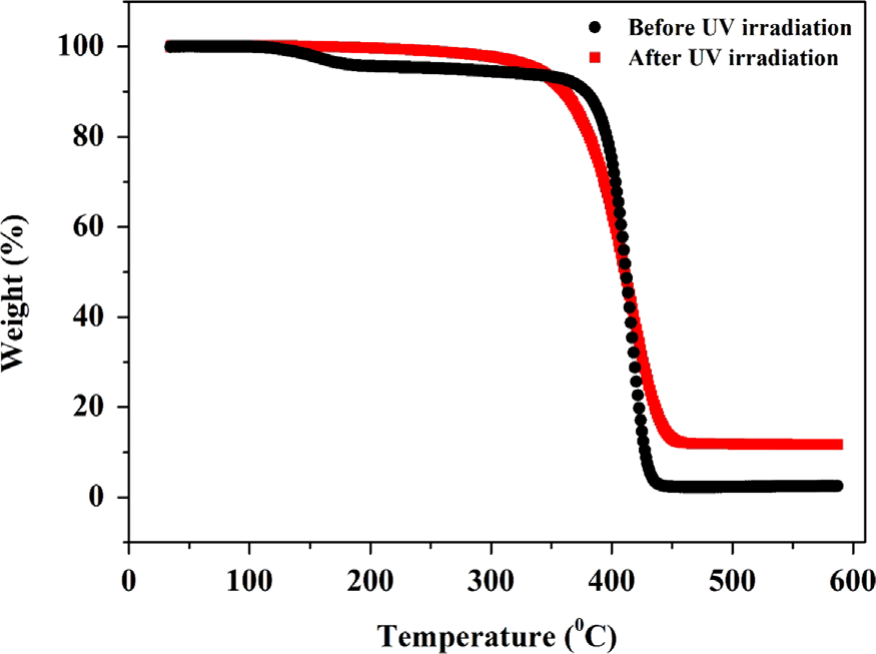
TGA plot of the PS honeycomb films before (black) and after (red) the 6 h UV irradiation.

The thermal property of the honeycomb films before and after applying UV treatment was characterized using TGA, as shown in Fig. 3. The as-prepared honeycomb PS thin film possesses both a glass transition temperature (~110 °C) and the decomposition temperature, while the crosslinked film shows only decomposition temperature. This is a reason for the reservation of honeycomb structure after annealing crosslinked film at 250 °C.

**Fig. 4:**
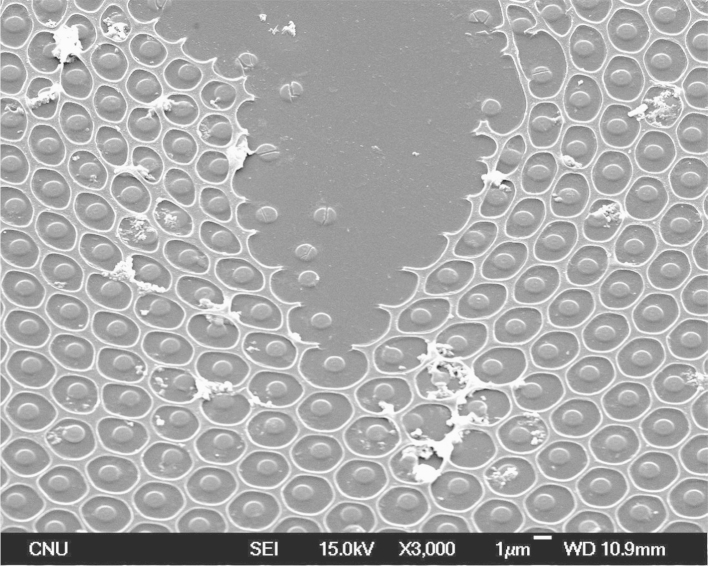
FESEM image of the annealed honeycomb film at a tear position.

For more insight into “disc-in-pore” structure after annealing, a 45° tilted SEM image of “disc-in-pore” film at a tear position is shown in Fig. 4. Discs with an average diameter of approximately 900 nm were located at the center of the pores and were separated from the honeycomb matrix by a gap of approximately 700 nm. This implies that the wafer-thin bottom layer inside the pore might be shrinked to form the “disc” at the center of the pore.

From a macroscopic perspective, the apparent contact angle was typically used to qualify the surface wettability, which is governed by the surface roughness and surface chemistry. The surface morphology of honeycomb film is almost not changed after UV treatment. Thus, the change of water contact angle after applying UV irradiation can be assigned to the change of surface chemistry. Table 1 shows the water contact angle of the PS honeycomb films before and after the 6 h UV irradiation. After UV irradiation, the water contact angle decreases considerably. It means that polar groups were introduced on the surface of honeycomb film after UV irradiation. The improvement of wettability of honeycomb film is favorable for the further applications such as cell culture and bio-sensors.

## Figures and Tables

**Table 1:** Water contact angle of the PS honeycomb films before and after the 6 h UV irradiation.

Sample	Water contact angle (deg)
Before UV irradiation	118±2.1
After UV irradiation	72±1.5
